# Experimental Manipulations to Test Theory-Driven Mechanisms of Cognitive Behavior Therapy

**DOI:** 10.3389/fpsyt.2020.603009

**Published:** 2020-12-17

**Authors:** Matthew W. Southward, Shannon Sauer-Zavala

**Affiliations:** Department of Psychology, University of Kentucky, Lexington, KY, United States

**Keywords:** cognitive behavior therapy (CBT), mechanism, SMART, personalization, unified protocol (UP)

## Abstract

Despite decades of randomized-controlled trials demonstrating the efficacy of cognitive-behavior therapy (CBT), the mechanisms by which CBT achieves its effects remain unclear. Here, we describe how one adaptive intervention, the sequential multiple assignment randomized trial (SMART), can be used to randomize patients at multiple decision points in treatment to draw stronger causal claims about mechanisms unfolding in the course of CBT. We illustrate this design using preliminary data and case examples from an ongoing SMART in which we are testing the role of aversive reactions to negative emotions as a hypothesized mechanism of change in the Unified Protocol. Finally, we address common concerns with SMARTs and highlight how mechanistic research serves to personalize and optimize the delivery of CBT.

## Introduction

Hundreds of randomized controlled trials (RCTs) have shown that cognitive behavior therapies (CBTs) are efficacious for many psychiatric conditions (1–3). RCTs are the optimal experimental design to test the efficacy of a treatment relative to comparison conditions. Randomly assigning patients to condition (e.g., CBT vs. waitlist) provides confidence that any post-treatment differences between conditions are due to the effects of the intervention(s), rather than patient characteristics or other confounding variables. RCTs for psychological treatments, particularly CBTs, were especially informative following *DSM-III* (4), which included more reliable symptom criteria for each psychiatric disorder. These criteria allowed researchers to conduct RCTs to assess how well CBTs addressed specific constellations of symptoms, relative to other treatment conditions.

Given the established efficacy of many CBTs (5), there has been an increased push to characterize which mechanisms drive symptom improvement [e.g., (6)]. Mechanisms of change are defined as “…core psychological and biological processes … [that] explain specifically how characteristics of the dysfunction are altered by the intervention and how that translates to symptom change” [(7), p. 87]. The relations among treatments, associated therapeutic mechanisms, and symptom change has often been explored statistically, whereby a mediator variable accounts for the relation between an intervention and an outcome (8–11). However, to determine whether a particular process constitutes a *mechanism* of symptom reduction, rather than simply a statistical mediator, several criteria must be met. These criteria include: (a) a strong association between the mechanism of action and the outcome, (b) temporal precedence where change in the mechanism precedes change in the outcome, (c) manipulating levels of the mechanism to determine how they relate to outcomes, and (d) a dose-response relation such that greater change in a mechanism leads to better outcomes (7, 9, 10).

Treatment researchers have made great strides toward identifying mechanisms of CBTs by utilizing more intensive measurements (e.g., at each session rather than only pre- and post-treatment). Frequent measurement of candidate mechanisms and psychiatric symptoms can provide evidence for criteria (a) and (b) for establishing a therapeutic mechanism. However, even when researchers statistically determine temporal precedence, results remain observational and can at best reveal Granger causality (12), which indicates that a temporally-lagged variable (*X*_*t*__−1_) explains unique variance in another variable at the next timepoint (*Y*_*t*_) above and beyond previous observations of that variable (*Y*_*t*−1_).

To draw stronger causal conclusions about the processes driving therapeutic improvements, it is necessary to apply innovative research designs that leverage the advantages of random assignment. We argue that sequential multiple assignment randomized trials [SMARTs; (13)], a type of multi-stage, experimental design developed for adaptive interventions, are an elegant way to evaluate treatment effects and mechanisms within a single clinical trial. We will first provide an overview of SMARTs and then describe how they can be adapted to evaluate the hypothesized mechanism of an intervention. We will present illustrative data from a SMART we are currently conducting to highlight how this design can provide a stringent, experimental test of core mechanisms.

## Sequential Multiple Assignment Randomized Trials (SMARTs)

SMARTs are a framework for evaluating adaptive interventions in clinical trials. In contrast to traditional RCTs that involve one primary clinical decision (e.g., randomizing a patient to the treatment or control condition), SMARTs contain multiple randomizations. For example, Chronis-Tuscano et al. (14) conducted a SMART to characterize best practices for families in which mothers and their children exhibit ADHD symptoms. In the initial randomization, mothers received stimulant medication or behavioral training to test which treatment approach is relatively more efficacious to start with. The second randomization occurred 8 weeks later; patients were randomized to either continue initial treatment or receive the alternative intervention as a supplement to their initial treatment. Because patients are randomized to receive treatment adaptations, SMARTs enable researchers to draw stronger conclusions about optimal treatment planning decisions.

In addition to randomizations based solely on time (e.g., re-randomizing all patients at session eight), researchers may also use *tailoring variables* to determine whether to adapt treatment. For instance, patients whose anxiety symptoms do not a reach a pre-determined threshold by a particular point in treatment may be re-randomized to continue with current care or receive more intensive treatment.

## Evaluating Mechanisms of Action Using SMARTs

Researchers implementing SMART designs are not limited to using symptoms as tailoring variables. In fact, we argue that to test hypothesized mechanisms of change in CBT, researchers should use the engagement of these mechanisms to guide treatment decision-making. Because changes in mechanisms should precede symptom changes, improvement in hypothesized mechanisms may represent an early indicator of eventual response. In this section, we describe the design of a pilot SMART we are currently conducting to evaluate methods for personalizing the delivery of the Unified Protocol [UP; (15)], an efficacious transdiagnostic CBT for a variety of psychiatric disorders (16, 17). In our initial randomization, patients with primary anxiety, depressive, or related disorders without imminent suicidal ideation are randomized to receive the modules (i.e., skills) from the UP in a personalized or standardized order. The second stage randomization occurs at mid-treatment (i.e., after 6 sessions), with patients assigned to either discontinue care immediately or receive the remaining six sessions. Patients randomized to discontinue immediately are sent weekly symptom measures to track their progress and are offered referrals as requested at the Week 12 follow-up assessment.

The developers of the UP have articulated a functional model of mood, anxiety, and related disorders in which these disorders are maintained by the transaction of frequent negative emotions (i.e., neuroticism) and aversive reactions to these emotions (18, 19). Aversive reactions may take many forms, including experiential avoidance, emotional suppression, worry, rumination, or distraction – any behavior used to escape or distract from one's emotions. Recent evidence suggests that reductions in certain forms of aversive reactions (e.g., experiential avoidance, anxiety sensitivity) precede and predict reductions in anxiety symptoms in CBT [(20–25); cf. (26)]. To measure aversive reactivity, participants in our SMART are completing the Distress Aversion subscale of the Multidimensional Experiential Avoidance Questionnaire [MEAQ-DA; (27)] before every weekly therapy session. The MEAQ-DA is a 13-item self-report measure designed to assess negative evaluations of and attitudes toward distress that has demonstrated good internal consistency across clinical samples (23, 27). The MEAQ-DA is sensitive to change in response to CBT for anxiety disorders [*d* = 0.82; (23)] and scores can range from 13–78.

We contend that our SMART design and, in particular, our secondary randomization (i.e., early termination vs. full course of care) are well-suited to evaluate aversive reactivity as a mechanism of change in the UP. The burgeoning evidence across independent treatment studies suggests the UP leads to changes in aversive reactivity. Variability in these changes indicate the degree to which the UP naturalistically manipulates different levels of this mechanism. Because patients are then randomized to receive 6 or 12 sessions, we can determine the degree to which aversive reactivity must improve in early sessions to predict maintenance or continued symptom improvement for patients who terminate at mid-treatment. Adequate symptom reduction at week 12 follow-up in patients who discontinue after six sessions *and* demonstrate mechanism engagement provides clear evidence for the importance of targeting aversive reactivity. However, unlike symptom measures, which have established threshold scores to determine patient progress, “mechanism engagement” for aversive reactivity has not been operationally defined. Thus, thresholds indicating the degree of change in mechanisms that predicts continued symptom improvement must first be established for measures of hypothesized mechanisms of treatment. Our current SMART will allow us to operationally define adequate target engagement of aversive reactivity as measured by the MEAQ-DA, allowing us to use these results to define the bounds of a tailoring variable in subsequent SMARTs. Randomizing patients to discontinue treatment after achieving a pre-specified cutoff for target engagement provides a more stringent test of whether candidate mechanisms are associated with downstream symptom improvement.

In the following section, we will present illustrative data from this trial as an example of how to establish target engagement thresholds by examining: (a) variability in MEAQ-DA scores; (b) whether early session changes in MEAQ-DA scores precede later session symptom changes; and (c) the magnitude of change on MEAQ-DA scores needed in early sessions to predict maintenance or continued improvement in symptoms for patients who terminate at mid-treatment.

### Variability in Hypothesized Mechanisms

Variability in our hypothesized mechanism, MEAQ-DA scores, is assessed in two ways: within each decision point and from one decision point to the next. Variability within a decision point is necessary to ensure that all participants would not be assigned to the same decision condition. If all patients had the same MEAQ-DA scores at mid-treatment, we would not be able to use this variable to make discontinuation decisions. Variability from one decision point to the next is necessary, in this case, to ensure the hypothesized mechanisms of change are themselves responsive to the study treatment. In our sample to date (*n* = 46), we have found substantial variability at both pre- (*M* = 45.00, *SD* = 11.06) and mid-treatment (*M* = 36.57, *SD* = 13.63) in the MEAQ-DA. Further, MEAQ-DA scores significantly decreased from pre- to mid-treatment, *t*(45) = −5.30, *p* < 0.01, 95% CI [−11.64, −5.23]. It is important to note, however, that without a control comparison group, these changes may, to some extent, indicate participant regression to the mean.

### Changes in Mechanisms Preceding Symptom Change

We selected the Overall Anxiety Severity and Impairment Scale [OASIS; (28)] as our measure of symptoms. Like the MEAQ-DA, the OASIS was administered at pre-, mid-, and post-treatment. In preliminary analyses of relatively smaller samples, it can be useful to determine the proportion of the sample for whom changes in hypothesized mechanisms precede changes in symptoms. Decreases in MEAQ-DA scores from pre- to mid-treatment preceded decreases in OASIS scores from mid- to post-treatment for 18 participants (39%) and increases in OASIS scores for 9 participants (20%). Similarly, decreases in OASIS scores from pre- to mid-treatment preceded decreases in MEAQ-DA scores for 20 participants (43%). These findings suggest that reductions in MEAQ-DA scores tend to precede later anxiety symptom improvements and not deterioration, although anxiety symptom improvement may also precede mechanism change for a substantial number of participants. Although these preliminary results provide mixed evidence for aversive reactivity as the sole mechanism of action in the UP, they demonstrate (a) the importance of comparing alternative hypotheses in the same study (10) and (b) the potential to identify moderators to distinguish the patients for whom aversive reactivity or anxiety symptoms function as mechanisms of change. When possible, researchers conducting experimental manipulations of treatment mechanisms should include competing experimental conditions or measures of alternative mechanisms, as in our SMART, that can be compared to researchers' primary theorized mechanism to provide a more stringent and comprehensive test of the hypothesis.

### Degree of Engagement in Hypothesized Mechanisms

Determining how much change is needed to consider a hypothesized mechanism engaged remains an open question. Researchers may choose relatively conservative but standardized metrics such as the reliable change index (29) as the standard of mechanism engagement. Alternatively, they may estimate the degree of change preceding certain symptom outcomes in one sample and apply this estimate to an independent hold-out sample. Given the preliminary stage of our study, we will highlight three exemplar cases of mechanism engagement and downstream clinical outcomes.

#### Patient 1

**Figure 1:**
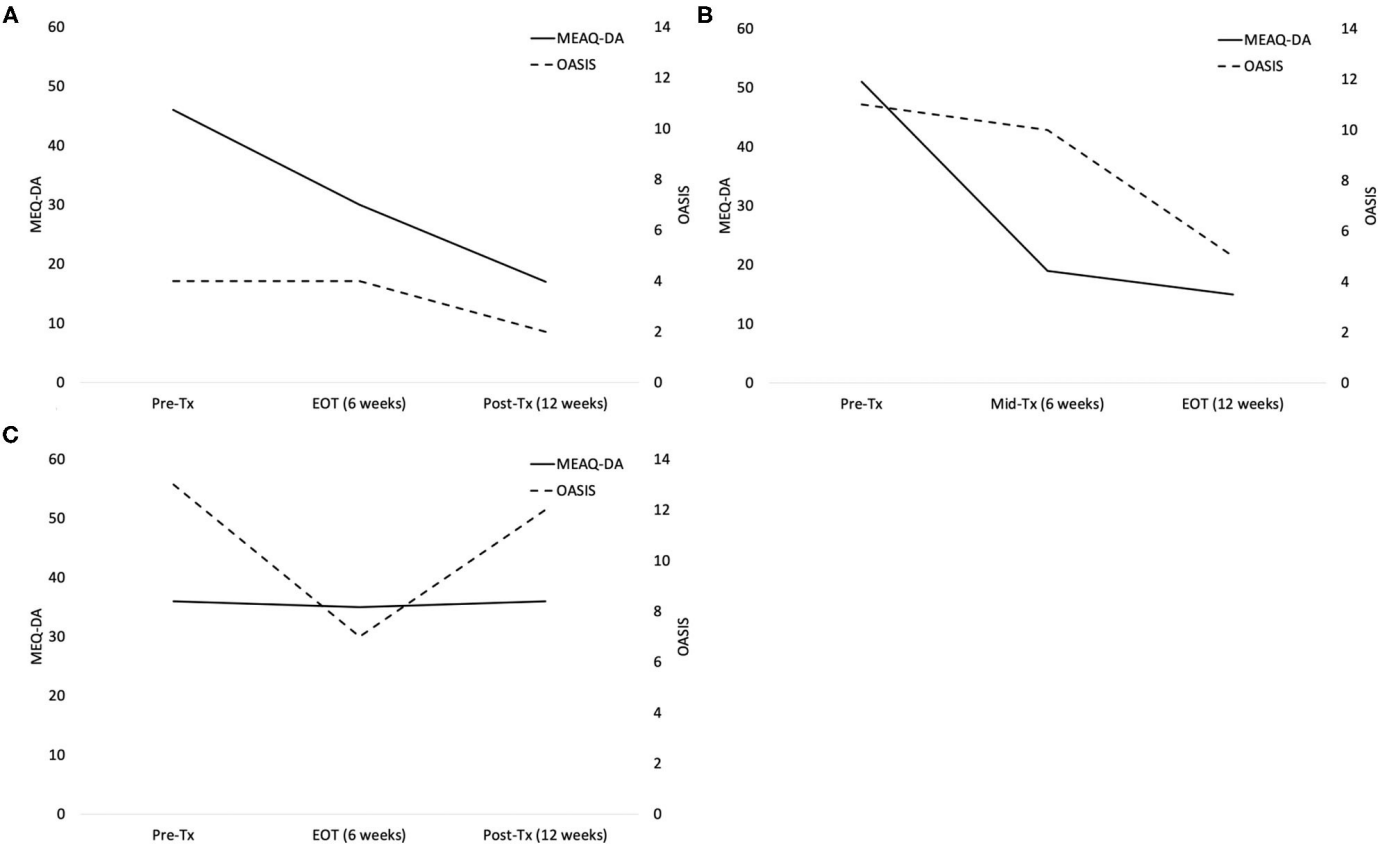
**(A)** Patient 1 MEAQ-DA and OASIS data demonstrating decreases in MEAQ-DA scores from pre-treatment (PreTx) to end-of-treatment (EOT) and subsequent decreases in OASIS scores following treatment discontinuation from EOT to post-treatment (Post-Tx). **(B)** Patient 2 MEAQ-DA and OASIS data demonstrating greater decreases in MEAQ-DA scores from Pre-Tx to mid-treatment (Mid-Tx) followed by greater subsequent decreases in OASIS scores from Mid-Tx to EOT. **(C)** Patient 3 MEAQ-DA and OASIS data demonstrating decreases in OASIS scores from Pre-Tx to EOT without corresponding decreases in MEAQ-DA scores, followed by a return to Pre-Tx OASIS scores by Post-Tx.

Patient 1 is a 61-year-old White female with primary generalized anxiety disorder (GAD) and specific phobia. She received UP modules in the standard order and discontinued treatment after six sessions. Thus, Patient 1 received the modules Understanding Emotions (UE), Mindful Emotion Awareness (MEA), and Cognitive Flexibility (CF; 2 sessions each) before discontinuing treatment. She demonstrated reliable change (Reliable Change Index [RCI] = −2.68) in MEAQ-DA scores from pre-treatment (46) to end-of-treatment (EOT; 30). However, she reported only a 1-point decrease in OASIS scores from pre-treatment (6) to EOT (5). Six weeks after treatment discontinuation, she reported a 40% decrease in anxiety severity (OASIS = 3; Figure 1A). This pattern of data suggests that achieving reliable change on the MEAQ-DA may predict continued symptom improvement even after treatment is withdrawn.

#### Patient 2

Patient 2 is a 45-year-old Arab-American male with primary social anxiety disorder and GAD. He received UP modules in a personalized order and completed the full course of treatment. Thus, he received the modules Counting Emotional Behaviors (CEB) and UE in the first six sessions and MEA, CF, and Confronting Physical Sensations (CPS) in the last six sessions. He demonstrated reliable change on the MEAQ-DA (RCI = −5.35) from pre- (51) to mid- (19) treatment. Similar to Patient 1, Patient 2 demonstrated almost no change in anxiety severity from pre- (OASIS = 11) to mid- (OASIS = 10) treatment. Instead, after six more sessions, he also reported a 50% decrease in anxiety severity (OASIS = 5) at EOT (Figure 1B). Of course, because Patient 2 continued to attend sessions after he achieved reliable change on the MEAQ-DA, it is difficult to discern whether his symptoms would have continued to improve if treatment had been discontinued after session 6.

By contrast, some patients demonstrate symptom improvement before mechanism engagement. In traditional SMARTs that rely on symptom changes to make clinical decisions, this may indicate a patient is a good candidate for treatment discontinuation. However, symptom improvement without corresponding mechanism engagement may not be as durable.

#### Patient 3

Patient 3 is a 33-year-old White female with primary GAD and body dysmorphic disorder. She received UP modules in a personalized order and discontinued treatment after six sessions. Thus, she received the CEB and CPS modules. She demonstrated substantial improvement in anxiety severity from pre- (OASIS = 13) to mid-treatment (OASIS = 7). However, her MEAQ-DA scores were little changed from pre- (36) to mid- (35) treatment (RCI = −0.17). Six weeks after treatment discontinuation, Patient 3 reported anxiety scores similar to pre-treatment (OASIS = 12), suggesting her symptom gains in treatment were not as durable (Figure 1C). This pattern of results suggests that changes in aversive reactivity to emotions may be an important therapeutic mechanism in the UP.

Of course, these are illustrative cases selected to demonstrate how our second-stage randomization (i.e., discontinue after 6 sessions or continue for 12 sessions) can be used to examine aversive reactivity as a mechanism of symptom improvement. Data from our full sample will allow us to establish the degree to which MEAQ-DA scores must improve to predict continued symptom reduction among participants randomized to discontinue treatment early. These data will be used to establish the thresholds necessary to use MEAQ-DA scores as a tailoring variable in future projects.

## Discussion

As researchers and funding agencies shift from evaluating treatment outcomes to understanding the mechanisms by which treatments function, innovative trial designs are necessary. In particular, SMARTs allow for experimental manipulation of mechanisms within efficacy or effectiveness trials. Here, we have illustrated how our current SMART enables us to answer hypotheses about the timing and degree of change in a hypothesized mechanism needed for continued symptom improvement following treatment discontinuation. By examining different characteristics of mechanisms in treatment (e.g., variability, timing relative to symptom change, and degree of engagement), researchers can better characterize replicable and actionable mechanisms that can ultimately lead to more targeted interventions.

We have highlighted one current limitation of mechanistic SMARTs, namely the lack of a consensus definition of mechanism engagement. We believe this is appropriate, given the relatively nascent state of this research. However, it is essential that researchers first identify likely transtheoretical mechanisms of change and assess the degree of change necessary for a mechanism to be considered “engaged” by a patient. This degree of change will likely involve a range of values that vary based on individual differences, so we encourage researchers to pool resources when possible to collect these data. A second common limitation of SMARTs is the sample size needed to provide adequate statistical power. Given even two levels of randomization, it may appear that the sample sizes needed would be impossibly large. However, as Almirall et al. (30) note, researchers are rarely interested in testing differences among all randomization combinations. Instead, researchers should pre-specify which comparisons are of most interest and calculate the necessary sample size based on these comparisons. For instance, in our study, one comparison we will make is between participants randomized to continue or discontinue treatment, regardless of whether they received a personalized or standardized order of UP modules. Because we expect treatment discontinuation to exert a larger effect on outcomes than module ordering, we will collapse across participants in the personalization and standardization conditions to maximize our statistical power. Practically, we encountered no limitations in recruitment for this SMART, likely because all patients received some treatment immediately. We are currently replicating this study in a community mental health clinic to test the acceptability of discontinuing treatment to real-world providers, as this may be another limitation of SMARTs with treatment discontinuation.

We content that adaptive interventions, such as SMARTs, offer a promising way to personalize and optimize CBT. By characterizing which mechanisms are engaged by which treatment processes, how much change is needed in these mechanisms for a given patient, and when treatment can be reliably discontinued, these experimental designs can have a substantial influence on our understanding of core mechanisms of action in treatment. Rather than relying solely on symptom changes, which may be an unreliable indicator of progress, researchers can leverage experimental manipulations of treatment mechanisms to identify measures that clinicians can incorporate relatively easily into their practice to enhance the efficacy, efficiency, and accessibility of CBT (31).

## Data Availability Statement

The raw data supporting the conclusions of this article will be made available by the authors, without undue reservation.

## Ethics Statement

The studies involving human participants were reviewed and approved by University of Kentucky Nonmedical Institutional Review Board. The patients/participants provided their written informed consent to participate in this study. Written informed consent was obtained from the individual(s) for the publication of any potentially identifiable images or data included in this article.

## Author Contributions

MS performed the data analysis and drafted the manuscript. SS-Z provided critical revisions. Both authors developed the manuscript concept, study design, collected the preliminary data, and approved the final version of the manuscript for submission.

## Conflict of Interest

The authors declare that the research was conducted in the absence of any commercial or financial relationships that could be construed as a potential conflict of interest.
